# Antiviral properties of two trimeric recombinant gp41 proteins

**DOI:** 10.1186/1742-4690-3-16

**Published:** 2006-03-03

**Authors:** Delphine Delcroix-Genête, Phenix-Lan Quan, Marie-Gaëlle Roger, Uriel Hazan, Sébastien Nisole, Cécile Rousseau

**Affiliations:** 1 Institut Cochin, Department of Infectious Diseases, 22 rue Méchain, 75014 Paris, France, INSERM, U 567, CNRS, UMR 8104, Faculté de Médecine René Descartes, UMR-S 8104, 75014 Paris, France; 2 Mymetics Corporation, 14, rue de la Colombière, 1260 Nyon, Switzerland; 3 Protein'eXpert SA, 15, rue des Martyrs, 38027 Grenoble, France; 4 Université Paris 7-Denis Diderot, UFR de Biochimie, 2 Place Jussieu, 75251 Paris, France

## Abstract

**Background:**

As it is the very first step of the HIV replication cycle, HIV entry represents an attractive target for the development of new antiviral drugs. In this context, fusion inhibitors are the third class of anti-HIV drugs to be used for treatment, in combination with nucleoside analogues and antiproteases. But the precise mechanism of HIV fusion mechanism is still unclear. Gp41 ectodomain-derived synthetic peptides represent ideal tools for clarifying this mechanism, in order to design more potent anti-HIV drugs.

**Results:**

Two soluble trimeric recombinant gp41 proteins, termed Rgp41B and Rgp41A were designed. Both comprise the N- and C-terminal heptad repeat regions of the ectodomain of HIV-1 gp41, connected by a 7-residue hydrophilic linker, in order to mimic the trimeric fusogenic state of the transmembrane glycoprotein. Both recombinant proteins were found to inhibit HIV-1 entry into target cells in a dose-dependent manner. Rgp41A, the most potent inhibitor, was able to inhibit both X4 and R5 isolates into HeLa cells and primary T lymphocytes. X4 viruses were found to be more susceptible than R5 isolates to inhibition by Rgp41A. In order to elucidate how the trimeric recombinant gp41 protein can interfere with HIV-1 entry into target cells, we further investigated its mode of action. Rgp41A was able to bind gp120 but did not induce gp120-gp41 dissociation. Furthermore, this inhibitor could also interfere with a late step of the fusion process, following the mixing of lipids.

**Conclusion:**

Taken together, our results suggest that Rgp41A can bind to gp120 and also interfere with a late event of the fusion process. Interestingly, Rgp41A can block membrane fusion without preventing lipid mixing. Although further work will be required to fully understand its mode of action, our results already suggest that Rgp41A can interfere with multiple steps of the HIV entry process.

## Background

The discovery of powerful antiviral compounds in the 90's raised hopes for the eradication of human immunodeficiency virus (HIV). However, AIDS still remains a major health problem throughout the world and despite the considerable success of highly active antiretroviral therapy (HAART), the identification of novel targets for therapy is sorely needed [1,2]. Indeed, although current drugs succeed in decreasing and controlling viral replication, complete eradication of the virus is still out of reach [3,4]. The persistence of virus even after long periods of treatment mainly results from the presence of cellular reservoirs that contain transcriptionally competent latent viruses capable of producing new infectious particles after cellular activation [4-6]. These latently infected cells are a permanent source of virus that lead to a rebound of the viral load after interruption of HAART [3,7]. Furthermore, patients often stop treatment due to the onset of side effects and viral resistance often develops, making one or more of the drugs ineffective. It is now clear that an effective treatment against HIV will require the use of multiple drugs targeting different stages of the replicative HIV-1 cycle. In this context, HIV entry represents an attractive target, as it is the earliest event of the infection cycle [1,8].

HIV entry is a multistep process involving complex interactions between the viral envelope glycoproteins and receptor molecules expressed at the surface of target cells [9-11]. Envelope glycoproteins consist of trimers of two noncovalently associated subunits, gp120 and gp41, generated by the proteolytic cleavage of a precursor protein, gp160. Whereas the surface subunit, gp120, is responsible for the binding to cell surface receptors, CD4 and a chemokine receptor, the transmembrane glycoprotein, gp41, promotes the direct fusion of viral and cellular membranes, allowing the viral core to enter the cytoplasm of the target cell [9,11].

The ectodomain of gp41 contains a hydrophobic N-terminus, referred to as the fusion peptide [12], and two heptad repeat regions, N-HR and C-HR (also designated N36 and C34) located at the N- and C-terminal of the gp41 ectodomain, respectively [13,14]. The sequential binding of gp120 to the cellular receptors triggers conformational changes in gp41, which adopts a conformation known as the pre-hairpin intermediate state, leading to the insertion of the hydrophobic N-terminal fusion peptide into the membrane of the target cell. Subsequently, the N- and C-terminal heptad repeat segments fold in an antiparallel manner to create a six-helix bundle (6HB) composed of an internal trimeric coiled-coil of N-terminal helices surrounded by three C-terminal HR helices that pack into the grooves of the coiled coil [15-18]. This transition from the prehairpin intermediate state to the stable 6HB structure brings the viral and cellular membranes into close proximity and allows membrane fusion [19,20].

Synthetic peptides corresponding to the N-HR and C-HR of gp41 block fusion and viral infection by binding to the transiently exposed HRs of gp41 during conformational changes, thus preventing 6HB formation [21]. C-peptides are based on the gp41 C-HR sequence and target the N-HR [22,23], whereas N-HR derived peptides are believed to bind the C-HR [24,25]. Both N- and C-HR derived peptides are able to block gp41-induced fusion, but C-peptides are more potent inhibitors. T-20 (also known as DP-178, Fuzeon^® ^or Enfuvirtide) is a synthetic peptide corresponding to 36 conserved residues within C-HR. This peptide potently inhibits viral entry and membrane fusion of both laboratory-adapted strains and primary isolates of HIV-1 [26,27] and was the first HIV fusion inhibitor to be approved for treatment of HIV-1 infection (for a review, see [28]). This inhibitor is believed to prevent 6HB formation by binding to the N-HR of gp41 [21,29]. However, as for other anti-HIV agents, resistant strains emerge [26,30], underlining the need for additional HIV fusion inhibitors. Such inhibitors would represent ideal tools to further investigate the mechanisms involved in gp41-mediated fusion and may open new avenues for the development of anti-HIV drugs.

In this study, we present the design of two soluble gp41-derived trimeric recombinant proteins produced in *E. coli*, which were termed Rgp41A and Rgp41B. Each of these two proteins are constituted of an N-domain spanning the N-HR (N36) and a C-domain spanning the C-HR (C34), associated via a 7-residue linker. Both recombinant proteins fold spontaneously into trimers and inhibit HIV-1 entry into target cells in a dose-dependent manner. Rgp41A, the most potent inhibitor, was able to inhibit viral entry of both X4 and R5 isolates into HeLa-CD4 or HeLa-CD4-CCR5 cells or into primary T lymphocytes. However, as previously described for T-20 [31-33], Rgp41A is a more potent inhibitor against X4 viruses. In order to elucidate the mechanism by which Rgp41A interferes with HIV-1 entry into target cells, we further investigated its mode of action. We show that Rgp41A is able to bind gp120 but this binding did not seem to induce gp120-gp41 dissociation. Furthermore, we show that this inhibitor interferes with a late step of the fusion process, following the mixing of lipids. Together, our observations suggest that Rgp41A inhibits HIV-1 entry by acting at different stages of the entry process.

## Results

### Characterization of recombinant proteins

**Figure 1:**
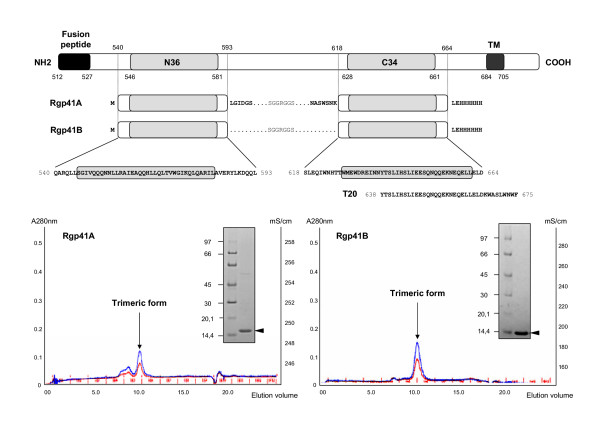
**Gp41-derived recombinant proteins**. A. Schematic representation of Rgp41A (N59(L7)C54) and Rgp41B (N54(L7)C47) synthetic trimeric peptides, derived from the HIV-1 gp41 ectodomain. Grey boxes represent the position of the N36 and C34 peptides. For comparison, the sequence of T20 is also indicated. B. Characterization of recombinant proteins. SDS-PAGE analysis and gel filtration on a Superdex 75 column. The elution profiles of Rgp41 proteins were compared to a calibration curve realized with standard globular proteins. An elution volume of 10.0 ml corresponds to an apparent molecular weight of 50 kDa.

Two recombinant proteins derived from the HIV-1 gp41 ectodomain were designed in order to mimic the trimeric fusogenic state of HIV-1 gp41 ectodomain and were referred to as Rgp41A and Rgp41B. Rgp41A comprises an N-domain of 59 residues, spanning the N-HR (or N36 peptide) and a C-domain of 54 residues, including the C-HR (or C34 peptide), whereas the N and C-domains of Rgp41B are 53 and 47 amino-acid long, respectively. Furthermore, thirteen (Rgp41A) or twenty five (Rgp41B) amino acids have been deleted between the N and C-domains, including the disulfide bridge. This gap was then replaced by an hydrophilic linker (_NH2_SGGRGGS_COOH_) for maintaining the N and C- domains connected. A 6xHIS tag was added at the C-terminal end of both constructs (LEHHHHHH) in order to allow their purification by immobilized metal ion affinity chromatography (IMAC). Figure 1A shows a schematic representation of both constructs. SDS-PAGE analysis of purified Rgp41A and Rgp41B revealed apparent molecular weights of 15 and 14 kD, respectively (Figure 1B). Both recombinant proteins were analyzed by gel filtration in order to determine their oligomeric state. As shown by their elution profiles on a Superdex 75 column, that corresponding to an apparent molecular weight of 50 kDa, both proteins appear to fold spontaneously into trimers. As expected, circular dichroism spectra of both trimeric recombinant proteins confirmed the presence of a high proportion of α-helix (not shown)[17,18].

### Recombinant gp41 proteins inhibit HIV-1 entry into CD4+ HeLa cells

**Figure 2:**
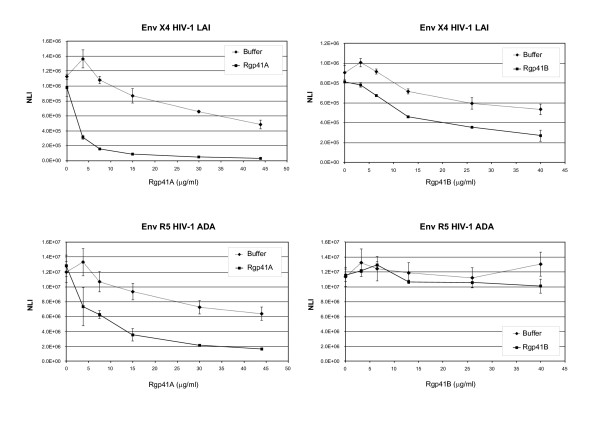
**Inhibition of HIV-1 entry into CD4+ HeLa cells by gp41-derived recombinant proteins**. HIV-1 particles pseudotyped with envelope glycoproteins from either the X4 isolate HIV-1 LAI or the R5 isolate HIV-1 ADA were pre-incubated with increasing concentrations of Rgp41A or Rgp41B before being added to HeLa-CD4-LTR-*LacZ *cells. For infection with HIV-1 ADA pseudotyped virus, cells were transfected with pCMV-CCR5 48 h before infection. Fourty-eight hours post-infection, viral entry and replication was monitored by measuring luciferase activity in cell extracts. For each experiment, the inhibitory effect of recombinant proteins was compared to the effect of the same volume of solubilization buffer. NLI: Normalized Luciferase Index. The average ± SD of triplicate samples is shown. Results represent the average ± SD of a typical experiment performed in duplicate, representative of at least 3 independent experiments.

Recombinant gp41 proteins were first tested for their ability to inhibit the infection of HeLa P4.2 cells by HIV-1 isolates. In the first set of experiments, we assayed the concentration-dependent inhibitory effect of Rgp41A and Rgp41B on HIV-1 particles pseudotyped with the envelope glycoproteins from the X4 isolate HIV-1 LAI or the R5 strain HIV-1 ADA. For this purpose, viruses were mixed with increasing doses of recombinant proteins prior to infection. Cells were incubated with the mixes for 4 h and rinsed several times to remove free viruses and recombinant proteins. After an incubation of 48 h at 37°C, virus replication was estimated by measuring luciferase activity in cell extracts. Since the buffers used for the solubilization of recombinant gp41 proteins showed some cytopathic effect, resulting in an artefactual decrease of the luciferase signal, the inhibitory effect of recombinant proteins was systematically compared to the same volume of buffer. Figure 2 shows the results of a typical experiment. Both constructs significantly inhibited entry of the X4 pseudotyped virus into host cells, whereas only Rgp41A has the capacity to also inhibit the entry of particles pseudotyped with R5 ADA Env. As expected, gp41-derived trimeric proteins had no effect on Vesicular Stomatitis Virus (VSV) envelope-pseudotyped viruses (not shown). IC50 values were calculated from these curves and reported in Table 1. Rgp41 showed IC50 values of 56 and 156 nM for HIV-1 LAI and ADA-pseudotyped viruses, respectively. Rgp41B has an IC50 value of 429 nM for LAI-pseudotyped virus. Similar experiments were performed on X4 laboratory-adapted viruses HIV-1 LAI and NDK or R5 HIV-1 YU2 and ADA. As for pseudotyped viruses, Rgp41A showed a better inhibitory effect than Rgp41B. The results are summarized in Table 1. Surprisingly, whereas Rgp41A showed an IC50 value of 156 nM on ADA pseudotyped HIV-1 particles, it displayed no inhibitory effect on the corresponding wild-type virus HIV-1 ADA. In contrast, Rgp41A inhibitory effects on the X4 strain HIV-1 NDK and the R5 strain HIV-1 YU2 were weak but significant, with IC50 values of 844 and 489 nM, respectively. Also shown for comparison in Table 1 are the IC50 values for T-20 on each virus. This inhibitor is approximately 25-fold more effective than Rgp41A to block HIV-1 LAI entry into HeLa-CD4 cells. Consistent with previous data, T-20 is not as effective on R5 isolates, such as YU2 and ADA, as on X4 viruses, such as HIV-1 LAI and NDK [31].

**Table 1:** IC50 values of Rgp41A and Rgp41B on the entry of pseudotyped or laboratory-adapted HIV-1 isolates into HeLa-CD4-LTR-LacZ cells.

		**Rgp41A**		**Rgp41B**		**T-20**	
		
		μg/ml	nM	μg/ml	nM	μg/ml	nM
**Pseudotyped**	LAI	2.5^a^	56	18	429	/	/
	ADA	7	156	NE	NE	/	/

**Laboratory-adapted**	LAI	13	289	42*	1000	0.05	11
	NDK	38*	844	70*	1667	0.04	9
	YU2	22	489	NE	NE	2	444
	ADA	NE^b^	NE	NE	NE	1.75	389

^a^These values correspond to the amount of recombinant proteins resulting in a 50% decrease in reporter activity compared to the value in the absence of inhibitor. Luciferase and β-Galactosidase were used as reporters of infection in the case of pseudotyped viruses and laboratory-adapted strains, respectively. This table shows the averages of a typical experiment performed in duplicate. *: Estimated from exponential regression analysis. ^b^NE:No effect.

### Rgp41A also blocks entry of X4 viruses into PBL

Antiviral properties of the Rgp41A were tested on the infection of PBLs by HIV-1 laboratory-adapted strains. Similar experiments were performed in parallel with T-20 (Table 2). In this model, the Rgp41A significantly blocked the entry of X4 viruses into host cells but had no effect on the R5 virus tested, suggesting that antiviral properties of the Rgp41A not only depend on the virus strain but also on the cell type. Calculation of IC50 values (Table 2) revealed that HIV-1 NDK was approximately 4 times more susceptible to inhibition by T-20 than by Rgp41A. IC50 values of Rgp41A on HIV-1 LAI and NDK are 356 and 322 nM, respectively.

**Table 2:** IC50 values of Rgp41A and T-20 on the entry of adapted HIV-1 isolates into PBL.

	**Rgp41A**		**T-20**	
	
	μg/ml	nM	μg/ml	nM
LAI	16^a^	356	/	/
NDK	14.5	322	0.38	84
YU2	NE^b^	NE	1.05	233

^a^These values represent the amount of recombinant proteins giving a 50% reduction of the amount of p24 in cell extracts, compared to the value in the absence of inhibitor and correspond to the mean value of a typical experiment. IC50 in nM correspond to the trimeric form of Rgp41A. ^b^NE: No effect.

### Rgp41A can interact with soluble monomeric gp120

**Figure 3:**
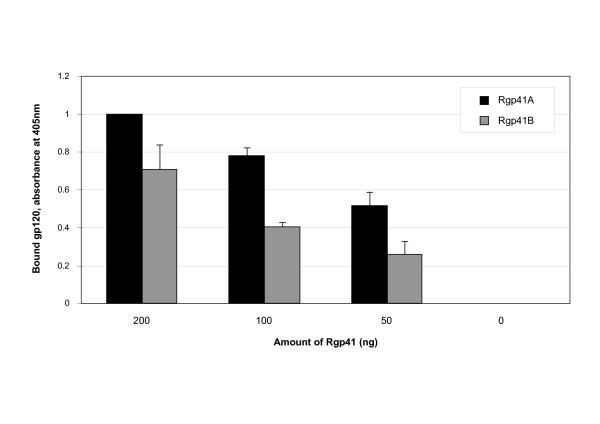
***In vitro *interaction between Rgp41 proteins and soluble monomeric gp120**. Ninety-six well plates were coated with increasing doses of recombinant gp41 proteins (0, 50, 100 or 200 ng) and then incubated with 2 ng of soluble gp120. Bound gp120 was revealed using specific anti-gp120 antibodies and HRP-conjugated secondary antibodies. Black bars: wells coated with Rgp41A, gray bars: wells coated with Rgp41B. Results represent the average of two independent experiments. Standard deviations are indicated by error bars.

In order to determine the mechanism of action of the most potent trimeric recombinant protein, Rgp41A, we first tested its ability to bind HIV-1 gp120. Some HIV-1 entry inhibitors act by binding to the envelope glycoproteins in order to interfere with their interaction with cellular receptors. This is the case for sCD4 and also for T-20, which was recently shown to interact with gp120 of X4 viruses, and to a lesser extent with gp120 of R5 viruses [33]. This interaction probably contributes to the mechanism by which T-20 blocks entry of X4 viruses into host cells [33]. Since both Rgp41 constructs contain the C34 sequence of T-20, we tested their ability to interact with a soluble monomeric recombinant gp120 from the X4 virus HIV-1_IIIB_. For this purpose, 96-well plates were coated with various doses of the Rgp41 proteins and then incubated with the monomeric gp120. The amount of gp120 bound to Rgp41 proteins was determined using anti-gp120 antibodies. As shown on Figure 3, the monomeric gp120 bound to both Rgp41 proteins in a dose dependent manner. Interestingly, Rgp41B retained significantly less gp120 than Rgp41A, suggesting a higher affinity of gp120 for Rgp41A than for Rgp41B.

### Rgp41A does not induce the release of gp120 from HeLa cells expressing HIV envelope

**Figure 4:**
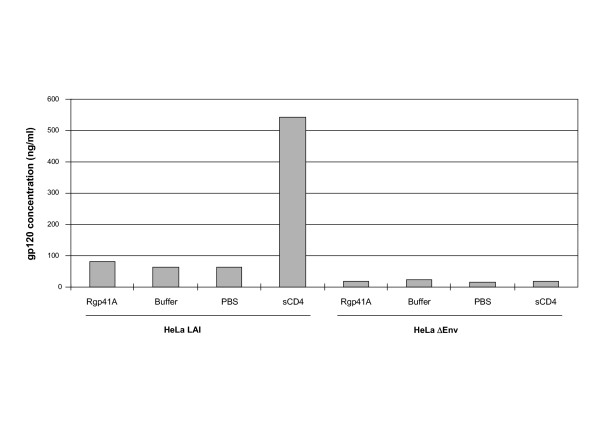
**Gp120 shedding**. HeLa-Env or HeLaΔ20 were incubated for 6 h at 37°C with Rgp41A (0.03 μg/μl), Rgp41A buffer (same volume), sCD4 or PBS. Release of gp120 from the cell surface was quantified by ELISA. Values represent averages of duplicate samples from a typical experiment.

Since recombinant gp41 appeared to be able to interact with a soluble monomeric gp120, we investigated whether this interaction could lead to gp120 release from the surface of the virus. Such a phenomenon has been reported for sCD4 and proposed to explain at least part of its antiviral properties [37-39]. To test gp120 shedding induced by recombinant gp41, HeLa cells expressing the *env *gene from the LAI virus (HeLa-LAI) were incubated with Rgp41A or sCD4. After incubation, the amount of gp120 present in the supernatant was measured by ELISA. As shown in Figure 4, Rgp41A did not induce the release of gp120 from the HeLa-LAI cells, in comparison to the control, whereas sCD4 induced the release of a significant amount of gp120. This result suggests that Rgp41A inhibits HIV entry into host cells by a mechanism that does not involve gp120 shedding.

### Rgp41A inhibits the fusion between cells expressing the *env *gene and target cells expressing HIV receptors

**Figure 5:**
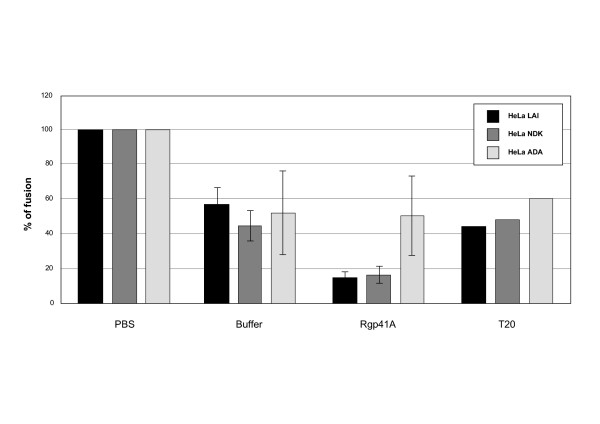
**Cell-to-cell fusion inhibition by the Rgp41A**. HeLa-Env/LAI, HeLa-Env/NDK, and HeLa-Env/ADA were incubated with target cells expressing the HIV receptors in the presence of PBS, Rgp41A (0.03 μg/μl), Rgp41A solubilization buffer or T-20 (0.2 μg/ml). Syncytia formation was evaluated by measuring the β-galactosidase activity after lysis of the cells. The results are expressed as percentage of the β-galactosidase activity observed in the control with PBS. Results represent the average of three independent experiments performed in duplicate. Standard deviations are indicated by error bars.

As cell-to-cell fusion experiments could be convenient models to analyze the mechanism by which Rgp41 proteins inhibit virus entry into host cells, we tested the efficacy of Rgp41A to inhibit the fusion between HeLa cells expressing the *env *gene of various HIV strains (HeLa-Env cells) and HeLa P4.2 cells expressing HIV receptors (target cells). For this purpose, HeLa-Env cells were incubated with Rgp41A prior to incubation with target cells, at a concentration that inhibits 90% of HIV-1 LAI infection (IC90). Cell fusion was monitored by measuring the β-galactosidase activity. As shown in Figure 5, the Rgp41A buffer appears to partially inhibit cell-to-cell fusion, probably reflecting its cytotoxicity. Indeed, we observed 45 to 55% reduction of β-galactosidase activity with Rgp41A solubilization buffer. In comparison with the buffer alone, Rgp41A inhibited nearly 4 times the fusion between HeLa-Env cells expressing the X4 HIV envelopes (LAI and NDK) and target cells, but had no significant effect on cells expressing a R5 envelope (ADA). Thus, in this model, Rgp41A activity seems to be restricted to the X4 HIV envelopes tested. For comparative purposes, we also included T20 in this experiment. At a concentration that inhibits 90% of HIV-1 LAI infection (0.2 μg/ml), T20 inhibits only around 50% of syncytia formation (Figure 5).

### Cell to cell fusion is inhibited by Rgp41A at a late stage during the fusion process

**Figure 6:**
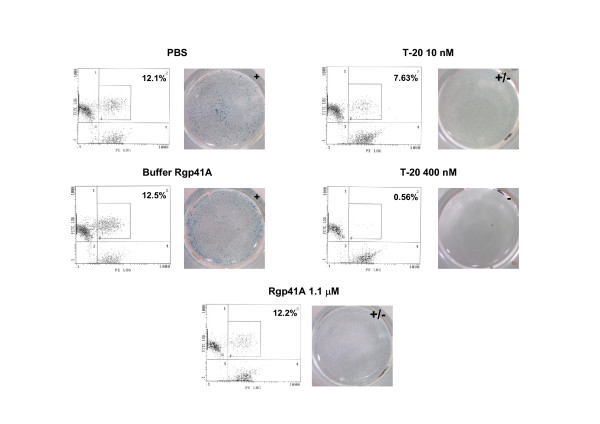
**lipid exchange and syncytia formation during cell-to-cell fusion experiments**. HeLa-Env/LAI and target cells were labeled with DiO and DiI, respectively. Co-cultures of labeled cells were performed in the presence of PBS (control), Rgp41A buffer, Rgp41A or T-20. After 6 h, lipid exchange between both types of cells was evaluated by flow cytometry analysis. In parallel, an X-Gal assay was performed to estimate the fusion efficiency between Env- and CD4-expressing cells in the presence or absence of inhibitors. Indicated percentages correspond to the proportion of double-positive gated cells. -: absence of syncytia, +/-: presence of small syncytia, +: presence of many large syncytia.

T-20 was recently shown to inhibit the membrane fusion process at a late stage, after the exchange of lipids between *env *expressing cells and target cells [35]. We investigated at which step of the fusion process Rgp41A acts. For this purpose, HeLa cells expressing the X4 LAI envelope and HeLa P4.2 target cells were labelled with two different hydrophobic fluorescent probes, DiO and DiI, respectively. Labelled HeLa-LAI cells were pre-incubated with Rgp41A, T-20, PBS or Rgp41A buffer, and then incubated with labelled target cells. After 6 h at 37°C, the amount of double fluorescent cells was measured by flow cytometry analysis. Double fluorescent cells result from an exchange of membrane lipids during the fusion process between HeLa-LAI and target cells. In parallel, the fusion efficiency was evaluated by measuring syncytia formation using an X-Gal assay. As shown in figure 6, the percentage of double fluorescent cells was about 12% when the cells were incubated with PBS, and the X-Gal assay showed the formation of many large syncytia, as expected. No significant difference was observed when the buffer of Rgp41A was used. At low dose (10 nM), T-20 had a limited effect on lipid exchange since about 7% of double fluorescent cells were observed, which corresponds to a reduction of about 37% of membrane exchange. However, it blocked syncytia formation, as shown by the small number and size of syncytia on the plate. In contrast, at higher dose (400 nM), T-20 has completely abolished syncytia formation and more than 95% of lipid exchange. Rgp41A significantly inhibited syncytia formation but did not inhibit the exchange of lipids, since the treatment of cells with Rgp41A did not significantly modify the amount of double fluorescent cells. Thus, these results suggest that Rgp41A, as for T-20 at low dose, inhibits the fusion process at a late stage after the mixing of lipids, since it appeared to block the formation of syncytia without preventing the exchange of lipids between HeLa-LAI cells and CD4-expressing cells.

## Discussion

In this study, two soluble trimeric HIV-1 gp41 recombinant proteins were shown to inhibit the HIV-1 fusion process. Both constructs comprise N and C domains connected by a 7-residue hydrophilic linker and were shown to fold spontaneously into a trimer, confirming that these proteins may mimic the six-helix bundle of the gp41 ectodomain in its fusogenic state [15-18,40]. Lu et al. previously described a gp41-derived construct named N34(L6)C28, formed by the gp41-derived N34 and C28 peptides associated by a 6 residue linker. This protein, which has the same overall structure as Rgp41B (N53(L7)C47) and Rgp41A (N59(L7)C54), was found to form highly thermostable α-helical trimers [41].

Infection experiments with pseudotyped or wild-type HIV-1 viruses on HeLa-CD4 or HeLa-CD4-CCR5 cells revealed that both Rgp41A and Rgp41B have the capacity to interfere with HIV-1 entry into target cells. It should be noted that both Rgp41 trimeric proteins have the propency to aggregate in solution, especially Rgp41A, presumably because of the presence of the N-HR [16]. The high degree of insolubility of Rgp41A lead us to use a solubilization buffer that was found to be toxic for the cells. Therefore, Rgp41A apparent effect on HIV-1 entry was systematically compared to the effect of its solubilization buffer alone.

The most potent inhibitor is Rgp41A, which inhibits the infection of HeLa-CD4 cells by HIV-1 LAI and ADA Env pseudotyped viruses with IC50 values of 56 and 156 nM, respectively. Its efficacy on wild-type viruses appeared weaker, with IC50 values from 289 nM for HIV-LAI to 844 nM for HIV-1 NDK. For comparison, N34(L6)C28 IC50 value on HIV-1 IIIB infectivity is 1.5 μM [42], indicating that the Rgp41A is about 5 times more efficient than N34(L6)C28 to inhibit X4 viruses infection.

In contrast, Rgp41A displayed no effect on the R5 strain HIV-1 ADA. Interestingly, HIV-1 ADA has been previously reported to be particularly resistant to different entry inhibitors [43,44]. This HIV-1 isolate appears therefore to behave differently compared to other known HIV-1 isolates for a reason that remains to be investigated. More generally, R5 isolates appeared less susceptible to Rgp41A inhibition than X4 strains. This higher sensitivity of X4 viruses has been previously observed with the C-HR derived fusion inhibitor, T-20 [31,32]. The second construct, Rgp41B, which contains shorter N and C domains, inhibits poorly X4 HIV-1 isolates LAI and NDK, at micromolar concentrations, and is inactive against R5 strains.

We next examined the mechanism by which Rgp41A interferes with HIV-1 entry into target cells. We demonstrate that the trimeric recombinant protein was able to interact with monomeric gp120 derived from an X4 HIV-1 isolate. However, this binding does not seem to result in the release of gp120, a mechanism partly responsible for inhibition of infection by sCD4 [37-39]. It may thus be possible that Rgp41A can bind preferentially to gp120 of X4 viruses, preventing its interaction with CD4 and/or CXCR4. This hypothesis would explain its better efficacy against X4 viruses entry, although this has yet to be fully investigated. Interestingly, the fusion inhibitor T-20 was also shown to bind gp120 of X4 HIV-1 strains in a CD4-induced, V3 loop dependent manner [33]. This binding was shown to prevent the interaction of gp120-CD4 complexes with the CXCR4 coreceptor.

In order to determine whether Rgp41A can affect different steps of the entry process in a similar way as T-20 [33,45], we tested the ability of Rgp41A to block the fusion process. As expected, the trimeric molecule revealed its capacity to potently inhibit the fusion between X4 Env-expressing cells and target cells expressing CD4 and CXCR4. This inhibition occurs at a late stage of this process, as revealed by the incapacity of Rgp41A to prevent membrane exchanges, even at high concentrations that efficiently block the formation of syncytia. Once again, this observation is reminiscent of previous results described for T-20 at low concentrations [35,46,47]. T-20 is believed to act by binding to the transiently exposed triple-stranded coiled-coil of NH2-terminal helices, thus preventing the 6-HB formation. This mechanism of action is in agreement with the finding that the fusion pore forms before the folding of the 6HB has been completed [48]. Unlike T-20 and other previously described fusion inhibitors, which are small gp41-derived peptides capable of binding to the transiently exposed HRs of gp41, Rgp41A is a rather large molecule (approximately 50 kD). The expected conformation for Rgp41A is a trimer of hairpins, mimicking the fusogenic conformation of HIV-1 gp41. Whether its large size allow the trimeric protein to gain access to gp41 during its conformational changes remains to be elucidated. However, the fact Rgp41A inhibits fusion without any effect on the lipid-mixing suggest it might also interfere with the 6HB formation.

N34(L6)C28 has also been found to inhibit HIV-1 Env-mediated membrane fusion, in agreement with our results [41]. Interestingly, the potency of these trimeric HR1-HR2 proteins to inhibit HIV-1 entry appears proportional to the the length of the N- and C-terminal domains, the less and most potent inhibitor being N34(L6)C28 and Rgp41A, respectively [41]. Synthetic peptides corresponding to the N-HR and C-HR of gp41 block fusion by binding to the transiently exposed HRs of gp41 during conformational changes, thus preventing 6HB formation [21]. C peptides are potent inhibitors of HIV-1 infectivity with activity at nanomolar concentrations, whereas N-peptides are relatively poor inhibitors, presumably due to their tendency to aggregate in solution [16]. Many groups have tried to design more potent inhibitors by combining multiple HR1 and HR2, such as N(CCG)-gp41 (HR1-HR1-HR2)[49], 5-Helix (HR1-HR2-HR1-HR2-HR1) [25], HR121 (HR1-HR2-HR1)[41], HR212 (HR2-HR1-HR2)[41] or other N-peptides-derived inhibitors such as IQN17 or IQN23 [24]. Although some of these constructs have a strong inhibitory effect, their precise mode of action is still unclear.

In the case of Rgp41A and B, the only difference between the two trimeric molecules is the lenght of the N- and C-terminal gp41-derived domains, which differ by only 6 and 7 residues, respectively. In consequence, it would be interesting to explain the reason why the inhibitory effect of Rgp41A on HIV entry is systematically much higher than its B counterpart, despite the fact they both have the same overall structure. In this context, the synthetis of intermediate constructs containing N- and C-terminal domains of increasing lenghts would be particularly informative in order to identify the determinants of this difference of antiviral activity.

## Conclusion

Both Rgp41 proteins were found to inhibit HIV-1 entry into target cells in a dose-dependent manner. Rgp41A, the most potent inhibitor, was found to inhibit both X4 and R5 isolates into HeLa cells and primary T lymphocytes. Rgp41A was able to bind gp120 but did not induce gp120-gp41 dissociation. Furthermore, this inhibitor interferes with a late step of the fusion process, following the mixing of lipids.

Considering our results, it is also possible that Rgp41A, like T-20, may act at different stages of the entry process. Although the precise mechanism of action of these HIV entry inhibitors will be difficult to unravel, it will undoubtedly help to elucidate the complex mechanisms involved during HIV entry process.

## Materials and methods

### Cell lines and plasmids

HeLa-CD4-LTR-*LacZ *(also referred as HeLa P4.2) cells stably express the human CD4 molecule and contain the β-galactosidase encoding gene (*lacZ*) under the transcriptional control of the HIV-1 long terminal repeat (LTR). They were kindly provided by Dr M. Alizon (Institut Cochin, Paris, France). HeLa-Env/ADA (or HeLa-ADA) cells stably express the envelope of the R5 tropic HIV strain ADA. HeLa-Env/LAI (or HeLa-LAI) and HeLa-Env/NDK (or HeLa-NDK) cells stably express LAI and NDK *env *genes from X4 viruses LAI and NDK, respectively. HeLaΔ20 cells are derived from HeLa-Env/LAI cells and contain a deletion in the *env *gene. Both cell lines were kindly provided by Dr M. Alizon (Institut Cochin, Paris, France). HeLa-Env and HeLaΔ20 cells also stably express the Tat HIV protein. All adherent cell lines were grown in Dulbecco's modified Eagle's medium (DMEM, Invitrogen) supplemented with 5% fetal calf serum (Invitrogen), 50 U/ml penicillin, 50 μg/ml streptomycin (Invitrogen) and 2 mM glutamine (Invitrogen).

The pCMV-CCR5 plasmid (kindly given by Dr. T. Dragic, New York, USA) contains the CCR5 gene under the control of the CMV promoter. The proviral plasmid pNL4.3-Δenv-Luc contains the NL4.3 *env*-deleted provirus including the luciferase reporter gene inserted in the *nef *ORF [34]. The LAI and ADA8 expression plasmids harbor the LAI and ADA8 *env *genes, respectively, under the control of the HIV-1 LTR. The pEnv-VSV-G plasmid encoding VSV-G envelope was a gift from Dr. P. Sonigo (Institut Cochin, Paris, France). The pADA, pJRCSF and pYU2 proviral plasmids encode proviral genomes of R5 tropic viruses, whereas the pNL4.3 and pNDK proviral plasmids encode proviral genomes of X4 tropic viruses.

### Antibodies and chemical reagents

The sheep anti-gp120 monoclonal antibody D7324 (Aalto) was raised against a C-terminal peptide of the gp120. Sera from HIV+ patients were a gift from Professor J.C. Nicolas (Tenon hospital, Paris, France). HRP-coupled anti-human and anti-sheep antibodies were purchased from Caltag and DAKO respectively. The HRP substrate ABTS from Roche was used at a concentration of 1 mg/ml. The fluorescent hydrophobic probes DiO and DiI were purchased from Sigma Aldrich. The fusion inhibitor T-20 and the soluble CD4 (sCD4) were obtained through the NIH AIDS Research and Reference Reagent Program. The recombinant protein gp120 HIV-1_IIIB _was purchased from Advanced Biotechnologies Incorporated.

### Production and purification of soluble trimeric recombinant gp41 proteins

The trimeric recombinant protein Rgp41A and Rgp41B were provided by Protein'eXpert (Grenoble, France) and produced as follow. Briefly, HIV-1 gp41 sequences corresponding to Rgp41A and Rgp41B were cloned between the NdeI and the XhoI sites of the pET21b and pET20b expression vectors (Novagen), respectively, allowing the production of recombinant protein harboring a 6xHIS tag at their C-terminus. Competent *Escherichia coli *BL21(DE3) were transformed with each vector and grown in LB medium at 37°C until an absorbance of 0.6 at 600 nm was reached. The production of recombinant protein was then induced by adding 1mM IPTG. Two hours after induction, bacteria were harvested and lysed in protein buffer (50 mM Tris-HCl, 300 mM NaCl, pH 8) by sonication. The suspension was then centrifuged at 40 000 g for 30 min at 4°C to separate the soluble proteins (supernatant) from the insoluble proteins and cell debris (pellet). Rgp41 proteins were purified from supernatant by affinity chromatography using Chelating Sepharose™ Fast Flow (Amersham Biosciences) and eluted using elution buffer (50 mM Tris-HCl, 300 mM NaCl, imidazole 500 mM, pH 8). Rgp41A-containing fractions were pooled and dialyzed against 50 mM Tris-HCl, 200 mM NaCl, 200 mM imidazole, pH 8. Rgp41B-containing fractions were pooled and dialyzed against 50 mM Tris-HCl, 200 mM NaCl, pH 8. Purity of the recombinant proteins was checked by 12% SDS-PAGE. The oligomeric status of the recombinant protein was determined by gel filtration chromatography using Superdex 75 HR 10/30 (Amersham Biosciences). Columns were equilibrated and eluted with 50 mM Tris-HCl, 200 mM NaCl; 200 mM imidazole; 5% glycerol; pH 8 in the case of Rgp41A and with 50 mM Tris-HCl, 200 mM NaCl, 5% glycerol, pH 8 in the case of Rgp41B. The calibration curve was obtained with standard globular proteins.

Rgp41A and Rgp41B were patented by Mymetics Corporation (ref PCT/IB2004/002433).

### Production of HIV-1 pseudotyped and laboratory-adapted strains

Stocks of pseudotyped viruses were generated by co-transfecting HEK293 cells with the proviral plasmid pNL4.3-Δenv-Luc and one of the *env *encoding plasmids. Stocks of adapted laboratory viruses were obtained by transfecting HEK 293 cells with the proviral plasmids pADA, pYU2, pNL4.3 or pNDK. Forty eight hours after transfection, the supernatant containing viruses was filtered and virus stocks were titrated by p24 ELISA (Coulter).

### Production and activation of PBL

Peripheral blood mononuclear cells were isolated from human blood on Ficoll (Ficoll-Paque PLUS, Amersham Biosciences), washed several times in PBS, EDTA 0.3 mM and stored frozen in fetal calf serum supplemented with 10% DMSO. For peripheral blood lymphocytes (PBL) production, stocks were quickly thawed and washed in RPMI, 10% fetal calf serum. Cells were cultured in 6-well plates in RPMI, 10% fetal calf serum. PBL were activated with 5 μg/ml PHA (DIFCO) and, three days later, with 40 U/ml IL-2 (Proleukin, Chiron). After two weeks of IL-2 induced proliferation, cells were used for infection experiments.

### Cell infections

HeLa P4.2 transfected with pCMV-CCR5 or PBL were cultured in 48-well plates (about 5 × 10^4 ^cells per well). Prior to infection, fixed concentrations of pseudotyped or laboratory-adapted viruses were incubated for 15 min with a range of Rgp41 concentrations (from 0.0025 to 0.03 μg/μl) or with the same volume of Rgp41 specific solubilization buffer. After incubation, mixes were added to the cells. The amount of viruses added per well was equivalent to 10 ng of p24. Four hours post-infection, cells were washed several times to remove free viruses and recombinant proteins and cultured for 48 h. Viral infectivity was monitored by measuring the luciferase activity in cell lysates in the case of HeLa cells infections by pseudotyped viruses. For infections with laboratory adapted HIV-1 strains, infection was monitored by measuring the β-galactosidase activity in cell lysates. Finally, PBL infections were followed by measuring the amount of p24 in the supernatant.

### Cell-to-cell fusion assay

HeLa-Env cells were seeded in 48-well plates (10^5 ^cells per well) with either Rgp41A (0.03 μg/μl), Rgp41A buffer or PBS. Fifteen minutes later, target cells (HeLa P4.2 or HeLa P4.2 transfected with pCMV-CCR5) were added to the wells (10^5 ^per well) and co-cultures were incubated for 6 h at 37°C.

### Beta-galactosidase assay

Cells grown in 48-well plates were lysed in 200 μl of lysis buffer (60 mM Na_2_HPO_4_, 40 mM NaH_2_PO_4_, 10 mM KCl, 10 mM MgSO_4_, 2.5 mM EDTA, 1.25‰ NP40, 50 mM β-mercaptoethanol) for 10 min. An equivalent volume of reaction buffer (61.9 mM Na_2_HPO_4_, 18.1 mM NaH_2_PO_4_, 10 mM MgCl_2_, 10 mM β-mercaptoethanol, 6 mM chlorophenol-β-D-galactose) was then added to the lysate. Kinetics were performed by measuring the absorbance at 575 nm for 30 min. The β-galactosidase activity corresponds to the slope of the curve.

### Luciferase assay

Cells grown in 48-well plates were lysed in 200 μl of lysis buffer (25 mM Tris pH 7.8, 8 mM MgCl_2_, 2 mM DTT, 1% Triton X-100, 15% glycerol) before adding 100 μl of lysis buffer containing 0.25 mM luciferin and 1 mM ATP. Luciferase activity was measured on a Berthold Luminometer (Lumat LB9507).

### p24 titration

The p24 protein was titrated using the HIV-1 p24 Antigen Assay Kit (Coulter), according to the supplier's instructions. Briefly, infected cells or viral stocks were lysed in Triton X-100 and the lysates introduced into wells pre-coated with mouse anti-p24 monoclonal antibodies. Bound p24 was revealed using biotin-coupled human anti-HIV IgG followed by HRP-coupled streptavidin. HRP reaction was initiated by adding the HRP substrate into the wells and stopped 30 min later with the stopping buffer. The absorbance at 450 nm was determined. Purified p24 was used to generate standard curves.

### Interaction of Rgp41 proteins with monomeric gp120

Protein Immobilizer plates (EXIQON) were coated with Rgp41 proteins (200, 100 or 50 ng in 15 mM Na_2_CO_3_, 35 mM NaHCO_3 _per well). After overnight coating at 4°C, wells were washed several times with PBS-Tween (PBS 1×, 0.05% Tween 20), saturated with PBS containing 10% fetal calf serum for 2 h and washed again with PBS-Tween. Recombinant monomeric gp120 derived from HIV-1_IIIB _diluted in PBS-Tween was then added to the well (2 ng per well). Plates were incubated for 2 h at room temperature and washed several times with PBS-Tween. gp120 bound to Rgp41 was labeled with anti-gp120 antibodies D7324 diluted in PBS-Tween for 2 h at room temperature followed by HRP-conjugated secondary antibodies diluted in PBS-Tween for an additional hour. Plates were washed extensively with PBS-Tween. The HRP substrate was then added to the wells and the absorbance at 405 nm was measured 10 min later.

### gp120 release from HeLa-Env cells

HeLa-Env and HeLaΔ20 cells (4 × 10^6 ^cells per tube in 200 μl DMEM, 10% fetal calf serum) were incubated with Rgp41A (0.03 μg/μL), Rgp41A buffer, sCD4 (50 μg/ml) or PBS at 37°C. Six hours later, supernatants were harvested to quantify the amount of gp120 released. For this purpose, 96-well Protein Immobilizer plates (EXIQON) were coated overnight at 4°C with anti-gp120 antibodies D7324 diluted in 15 mM Na_2_CO_3_, 35 mM NaHCO_3_, pH 9.6. Wells were rinsed several times with PBS-Tween. Supernatants were then deposited into the wells and incubated for 2 h at room temperature. After several washes, a human anti-HIV serum was added into the wells and incubated for 2 h at room temperature. Wells were washed and HRP-coupled secondary antibodies diluted in PBS-Tween were added into the wells for an additional hour at room temperature. Wells were washed extensively before addition of the HRP substrate. The absorbance at 405 nm was measured 1 h after initiation of the reaction. Standard curves were obtained with purified HIV-1_IIIB _gp120.

### Lipid mixing analysis

HeLa-Env cells and HeLa P4.2 target cells were labeled with 2 μM DiO and DiI, respectively, as previously described [35]. After cell labeling, a cell-to-cell fusion assay was performed as described above, in the presence of Rgp41A (50 μg/ml), Rgp41A buffer, T-20 (10 nM or 400 nM) or PBS. Co-cultures were incubated for 6 h at 37°C. Cells were then detached from wells with PBS, 15 mM citrate, pH 7 and fixed with PBS 2% formaldehyde. Double fluorescent cells, containing both DiO and DiI, were detected by two color XL_2 _Beckman Coulter cytometer using the System II™ software. At least 10^4 ^cells were counted for each sample. In parallel, some cells were subjected to an X-Gal assay in order to estimate cell to cell fusion efficiency, as described previously [36].

## Competing interests

The author(s) declare that they have no competing interests.

## Authors' contributions

DDG carried out HIV infections, cell to cell fusion experiments, ELISA and lipid mixing assays. PLQ participated in HIV inhibition experiments and also in the experimental design and data analysis. MGR produced and performed the structural analysis of Rgp41 proteins. UH conceived of the study and participated in its design and coordination, as well as in the writing of the manuscript. SN participated in the data analysis and drafted the manuscript. CR participated in the experimental design and data analysis and also performed HIV infection and cell to cell fusion experiments. All authors have read and approved the final manuscript.

## References

[B1] Moore JP, Stevenson M (2000). New targets for inhibitors of HIV-1 replication. Nat Rev Mol Cell Biol.

[B2] Fauci AS (2003). HIV and AIDS: 20 years of science. Nat Med.

[B3] Chun TW, Davey RT, Engel D, Lane HC, Fauci AS (1999). Re-emergence of HIV after stopping therapy. Nature.

[B4] Finzi D, Blankson J, Siliciano JD, Margolick JB, Chadwick K, Pierson T, Smith K, Lisziewicz J, Lori F, Flexner C, Quinn TC, Chaisson RE, Rosenberg E, Walker B, Gange S, Gallant J, Siliciano RF (1999). Latent infection of CD4(+) T cells provides a mechanism for lifelong persistence of HIV-1, even in patients on effective combination therapy. Nature Medicine.

[B5] Pomerantz RJ (2002). Eliminating HIV-1 reservoirs. Curr Opin Investig Drugs.

[B6] Hamer DH (2004). Can HIV be cured? Mechanisms of HIV persistence and strategies to combat it. Curr HIV Res.

[B7] Chun TW, Fauci AS (1999). Latent reservoirs of HIV: obstacles to the eradication of virus. Proc Natl Acad Sci U S A.

[B8] Pierson TC, Doms RW (2003). HIV-1 entry and its inhibition. Curr Top Microbiol Immunol.

[B9] Wyatt R, Sodroski J (1998). The HIV-1 envelope glycoproteins: fusogens, antigens, and immunogens. Science.

[B10] Altmeyer R (2004). Virus attachment and entry offer numerous targets for antiviral therapy. Curr Pharm Des.

[B11] Clapham PR, McKnight A (2002). Cell surface receptors, virus entry and tropism of primate lentiviruses. J Gen Virol.

[B12] Gallaher WR (1987). Detection of a fusion peptide sequence in the transmembrane protein of human immunodeficiency virus. Cell.

[B13] Delwart EL, Mosialos G, Gilmore T (1990). Retroviral envelope glycoproteins contain a 'leucine zipper'-like repeat. AIDS Res Hum Retroviruses.

[B14] Chambers P, Pringle CR, Easton AJ (1990). Heptad repeat sequences are located adjacent to hydrophobic regions in several types of virus fusion glycoproteins. J Gen Virol.

[B15] Chan DC, Fass D, Berger JM, Kim PS (1997). Core structure of gp41 from the HIV envelope glycoprotein. Cell.

[B16] Lu M, Blacklow SC, Kim PS (1995). A trimeric structural domain of the HIV-1 transmembrane glycoprotein. Nat Struct Biol.

[B17] Tan K, Liu J, Wang J, Shen S, Lu M (1997). Atomic structure of a thermostable subdomain of HIV-1 gp41. Proc Natl Acad Sci U S A.

[B18] Weissenhorn W, Dessen A, Harrison SC, Skehel JJ, Wiley DC (1997). Atomic structure of the ectodomain from HIV-1 gp41. Nature.

[B19] Eckert DM, Kim PS (2001). Mechanisms of viral membrane fusion and its inhibition. Annu Rev Biochem.

[B20] Gallo SA, Finnegan CM, Viard M, Raviv Y, Dimitrov A, Rawat SS, Puri A, Durell S, Blumenthal R (2003). The HIV Env-mediated fusion reaction. Biochim Biophys Acta.

[B21] Chan DC, Kim PS (1998). HIV entry and its inhibition. Cell.

[B22] He Y, Vassell R, Zaitseva M, Nguyen N, Yang Z, Weng Y, Weiss CD (2003). Peptides trap the human immunodeficiency virus type 1 envelope glycoprotein fusion intermediate at two sites. J Virol.

[B23] Rimsky LT, Shugars DC, Matthews TJ (1998). Determinants of human immunodeficiency virus type 1 resistance to gp41-derived inhibitory peptides. J Virol.

[B24] Eckert DM, Kim PS (2001). Design of potent inhibitors of HIV-1 entry from the gp41 N-peptide region. Proc Natl Acad Sci U S A.

[B25] Root MJ, Kay MS, Kim PS (2001). Protein design of an HIV-1 entry inhibitor. Science.

[B26] Kilby JM, Hopkins S, Venetta TM, DiMassimo B, Cloud GA, Lee JY, Alldredge L, Hunter E, Lambert D, Bolognesi D, Matthews T, Johnson MR, Nowak MA, Shaw GM, Saag MS (1998). Potent suppression of HIV-1 replication in humans by T-20, a peptide inhibitor of gp41-mediated virus entry. Nat Med.

[B27] Wild CT, Shugars DC, Greenwell TK, McDanal CB, Matthews TJ (1994). Peptides corresponding to a predictive alpha-helical domain of human immunodeficiency virus type 1 gp41 are potent inhibitors of virus infection. Proc Natl Acad Sci U S A.

[B28] Matthews T, Salgo M, Greenberg M, Chung J, DeMasi R, Bolognesi D (2004). Enfuvirtide: the first therapy to inhibit the entry of HIV-1 into host CD4 lymphocytes. Nat Rev Drug Discov.

[B29] Kliger Y, Shai Y (2000). Inhibition of HIV-1 entry before gp41 folds into its fusion-active conformation. J Mol Biol.

[B30] Wei X, Decker JM, Liu H, Zhang Z, Arani RB, Kilby JM, Saag MS, Wu X, Shaw GM, Kappes JC (2002). Emergence of resistant human immunodeficiency virus type 1 in patients receiving fusion inhibitor (T-20) monotherapy. Antimicrob Agents Chemother.

[B31] Derdeyn CA, Decker JM, Sfakianos JN, Wu X, O'Brien WA, Ratner L, Kappes JC, Shaw GM, Hunter E (2000). Sensitivity of human immunodeficiency virus type 1 to the fusion inhibitor T-20 is modulated by coreceptor specificity defined by the V3 loop of gp120. J Virol.

[B32] Derdeyn CA, Decker JM, Sfakianos JN, Zhang Z, O'Brien WA, Ratner L, Shaw GM, Hunter E (2001). Sensitivity of human immunodeficiency virus type 1 to fusion inhibitors targeted to the gp41 first heptad repeat involves distinct regions of gp41 and is consistently modulated by gp120 interactions with the coreceptor. J Virol.

[B33] Yuan W, Craig S, Si Z, Farzan M, Sodroski J (2004). CD4-induced T-20 binding to human immunodeficiency virus type 1 gp120 blocks interaction with the CXCR4 coreceptor. J Virol.

[B34] Moore JP, McKeating JA, Weiss RA, Sattentau QJ (1990). Dissociation of gp120 from HIV-1 virions induced by soluble CD4. Science.

[B35] Hart TK, Kirsh R, Ellens H, Sweet RW, Lambert DM, Petteway SRJ, Leary J, Bugelski PJ (1991). Binding of soluble CD4 proteins to human immunodeficiency virus type 1 and infected cells induces release of envelope glycoprotein gp120. Proc Natl Acad Sci U S A.

[B36] Sattentau QJ, Moore JP (1991). Conformational changes induced in the human immunodeficiency virus envelope glycoprotein by soluble CD4 binding. J Exp Med.

[B37] Bar S, Alizon M (2004). Role of the ectodomain of the gp41 transmembrane envelope protein of human immunodeficiency virus type 1 in late steps of the membrane fusion process. J Virol.

[B38] Furuta RA, Wild CT, Weng Y, Weiss CD (1998). Capture of an early fusion-active conformation of HIV-1 gp41. Nat Struct Biol.

[B39] Lu M, Ji H, Shen S (1999). Subdomain folding and biological activity of the core structure from human immunodeficiency virus type 1 gp41: implications for viral membrane fusion. J Virol.

[B40] Ji H, Shu W, Burling FT, Jiang S, Lu M (1999). Inhibition of human immunodeficiency virus type 1 infectivity by the gp41 core: role of a conserved hydrophobic cavity in membrane fusion. J Virol.

[B41] Ghorpade A, Xia MQ, Hyman BT, Persidsky Y, Nukuna A, Bock P, Che M, Limoges J, Gendelman HE, Mackay CR (1998). Role of the beta-chemokine receptors CCR3 and CCR5 in human immunodeficiency virus type 1 infection of monocytes and microglia. J Virol.

[B42] Nisole S, Krust B, Callebaut C, Guichard G, Muller S, Briand JP, Hovanessian AG (1999). The anti-HIV pseudopeptide HB-19 forms a complex with the cell-surface-expressed nucleolin independent of heparan sulfate proteoglycans. J Biol Chem.

[B43] Liu S, Lu H, Niu J, Xu Y, Wu S, Jiang S (2005). Different from the HIV fusion inhibitor C34, the anti-HIV drug Fuzeon (T-20) inhibits HIV-1 entry by targeting multiple sites in gp41 and gp120. J Biol Chem.

[B44] Kliger Y, Gallo SA, Peisajovich SG, Munoz-Barroso I, Avkin S, Blumenthal R, Shai Y (2001). Mode of action of an antiviral peptide from HIV-1. Inhibition at a post-lipid mixing stage. J Biol Chem.

[B45] Munoz-Barroso I, Durell S, Sakaguchi K, Appella E, Blumenthal R (1998). Dilation of the human immunodeficiency virus-1 envelope glycoprotein fusion pore revealed by the inhibitory action of a synthetic peptide from gp41. J Cell Biol.

[B46] Markosyan RM, Cohen FS, Melikyan GB (2003). HIV-1 envelope proteins complete their folding into six-helix bundles immediately after fusion pore formation. Mol Biol Cell.

[B47] Louis JM, Bewley CA, Clore GM (2001). Design and properties of N(CCG)-gp41, a chimeric gp41 molecule with nanomolar HIV fusion inhibitory activity. J Biol Chem.

[B48] Connor RI, Sheridan KE, Ceradini D, Choe S, Landau NR (1997). Change in coreceptor use correlates with disease progression in HIV-1-infected individuals. J Exp Med.

[B49] Dumonceaux J, Nisole S, Chanel C, Quivet L, Amara A, Baleux F, Briand P, Hazan U (1998). Spontaneous mutations in the env gene of the human immunodeficiency virus type 1 NDK isolate are associated with a CD4-independent entry phenotype. J Virol.

